# Supplementary material for: Observation of two-dimensional Anderson localisation of ultracold atoms
Source: Nat Commun. 2020 Oct 2;11:4942. doi: 10.1038/s41467-020-18652-w (PMC7532155; doi:10.1038/s41467-020-18652-w)
Supplement: Supplementary file 1 — Supplementary Information [file 41467_2020_18652_MOESM1_ESM.pdf]

Supplementary Material — Observation of two-dimensional Anderson localisation of ultracold atoms

Donald H. White,[1,2,3] Thomas A. Haase,[1,2] Dylan J. Brown,[1,2,4] Maarten D. Hoogerland,[1,2] Mojdeh S. Najafabadi,[1,5] John L. Helm,[1,5] Christopher Gies,[6] Daniel Schumayer,[1,5] and David A. W. Hutchinson[1,5]

[1] *Dodd-Walls Centre for Photonic and Quantum Technologies, New Zealand*
[2] *Department of Physics, University of Auckland, Auckland, New Zealand*
[3] *Present address: Waseda Research Institute for Science and Engineering, Waseda University, Shinjuku, Tokyo, Japan*
[4] *Present address: Light-Matter Interactions for Quantum Technologies Unit, Okinawa Institute of Science and Technology, Tancha, Onna, Okinawa, Japan*
[5] *Department of Physics, University of Otago, Dunedin, New Zealand*
[6] *Institut für Theoretische Physik, Universität Bremen, Bremen, Germany*

Here we provide additional supporting information for the results described in the main text "Observation of two-dimensional Anderson localisation of ultracold atoms".

I. EXPERIMENTAL DETAILS

This experiment has been designed to be a 'quantum simulator' of 2D transport physics. This requires full knowledge of the topography of the 2D potential landscape. Ideally, the basic 2D trap would be flat, with disorder and boundaries introduced by the SLM-projected landscape. While we approximate this condition, we have found the presence of 'fringes' within the 2D trap. These fringes run along the y-direction, and have a period of $\approx 150\ \mu$m. The fringe depth is on the order of 5 nK. We attribute the fringes to the interference within the 1064 nm beams, which occurs from distortion to the phase-front of the beam from the vacuum window. The fringe central position may be adjusted by relative horizontal alignment of the two interfering 1064 nm beams, and we set the fringe centre to overlap the centre of the source reservoir.

The BEC is initially formed in a CO_2 crossed beam laser trap, and adiabatically loaded into the two-dimensional trap by ramping down the CO_2 laser power. Prior to the release of the CO_2 laser trap, the trap frequency in the horizontal directions is on the order of $\omega_{\rm x,y} \approx 2\pi \cdot 50$ Hz. The dimensionless interaction strength $\tilde{g} = a_{\rm s}\sqrt{8\pi m\omega_{\rm z}/\hbar} = 0.07$. The peak density within the CO_2 laser trap is $n_0 \approx \frac{m}{\hbar}\sqrt{\frac{N}{\pi\tilde{g}}}\omega_{\rm x,y} \approx 120$ atoms/μm^2. The healing length $\xi = 1/\sqrt{n_0\tilde{g}} = 350$ nm. The chemical potential within the CO_2 laser trap is $\mu = \hbar^2 n_0 \tilde{g}/m$, and $\mu/k_{\rm B} = 40$ nK. Once the atoms have been released from the CO_2 laser trap and expanded into the dumbbell, the density reduces to the order of 1 atom/μm^2, reducing the chemical potential to $\mu/k_{\rm B} = 0.3$ nK [1, 2].

A linear slope, with acceleration 0.002 m/s, is applied to the 2D trap, meaning that atoms are no longer bound by the fringe, and may enter the trap. While the presence of the fringe affects the bulk motion of the atoms within the channel, the period of the fringe is larger than the channel lengths used, and this means that the Anderson localisation physics should be unchanged. We note close agreement with GPE simulations (discussed below), even if the fringe pattern is excluded.

The BEC is placed such that its centre lies about half way between the centre of the source reservoir and the channel opening. We calibrate the acceleration by adjusting the tilt such that the first wave of atoms arrives at the far end of the drain reservoir at 100 ms after releasing the atoms from the dipole trap, using a 144 μm long channel.

II. FULL DATASETS

We include in Supplementary Figs. 1–6 the full datasets for a range of channel widths. The data indicates that steady-state exponentially-localised channel profiles are obtained for a broad range of channel widths in the presence of disorder. The exception to this is the 14 μm width channel, where the profile is non-exponential, and exhibits a non-uniform channel profile in the case of zero applied disorder. In this case, we are in a nearly one-dimensional regime, and minor channel disorder due to imperfections in the flat disordered potential results in localised eigenfunctions. In addition, there is significant reflection at the mouth of the channel due to the small channel opening, resulting in the dropoff in channel density seen at the channel opening.

III. WIDTH DEPENDENCE

Supplementary Figure 7 shows the localisation lengths extracted from the density profiles in Supplementary Figs. 4-6. For $w \lesssim 50\ \mu$m, the localisation length is significantly larger than the channel width and finite size effects are strong. For the quasi-2D environment of $w \gtrsim 50\ \mu$m, in which the localisation length is of the same order as the channel width, we show in Supplementary Fig. 7 that the width does not have a significant influence on the observed localisation length.

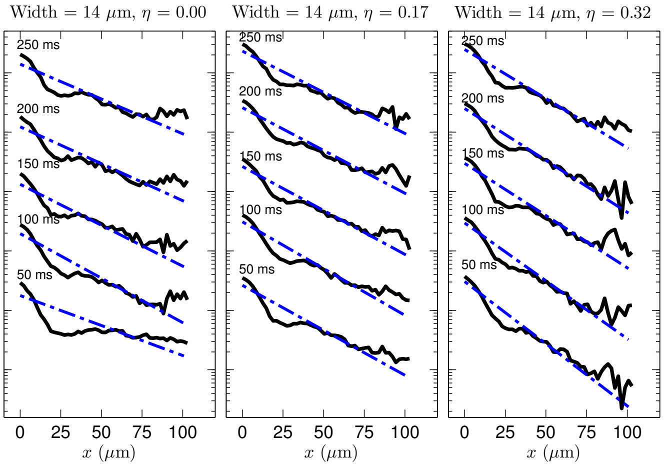

Supplementary Figure 1. 14 μm width channel density profile data. Channel profiles from 50 ms to 250 ms are plotted on a semilogarithmic scale, together with a linear fit to the logarithmic data. This data is an average of three experimental disorder realisations.

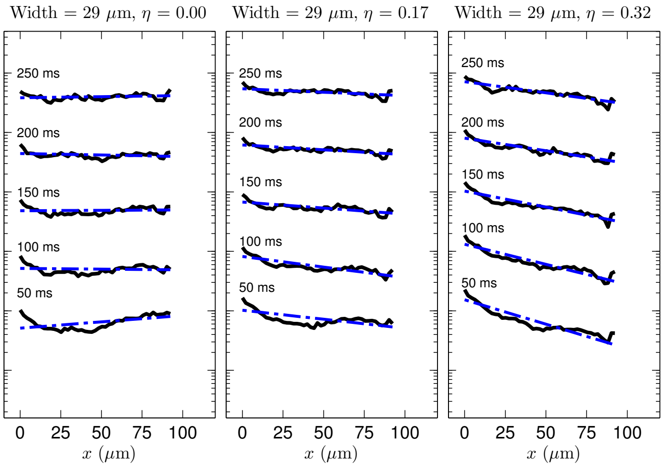

Supplementary Figure 2. 29 μm width channel density profile data. Channel profiles from 50 ms to 250 ms are plotted on a semilogarithmic scale, together with a linear fit to the logarithmic data. This data is an average of three experimental disorder realisations.

IV. SIMULATIONS FOR LONG TIMES

We show in Supplementary Fig. 8 simulations conducted for long times for the same conditions as Main Fig. 2. The data show that in the case of $\eta = 0.07$, a long-time exponential density profile is not obtained, but the density profile remains exponential for $\eta = 0.17$ and $\eta = 0.32$. We note that this exponential character remains, despite complications from higher energy atoms re-entering the channel from the drain reservoir.

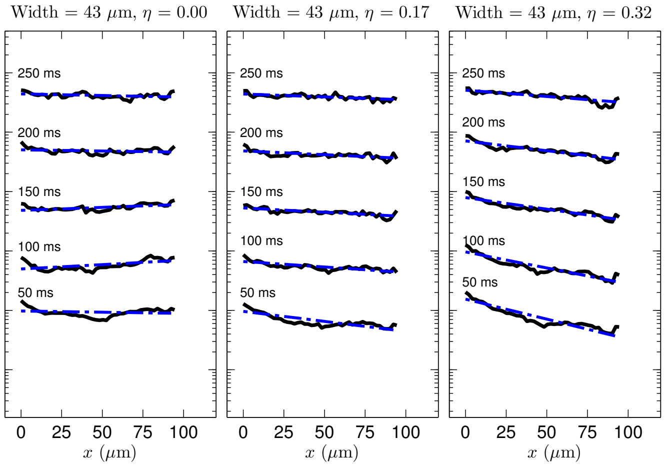

Supplementary Figure 3. 43 μm width channel density profile data. Channel profiles from 50 ms to 250 ms are plotted on a semilogarithmic scale, together with a linear fit to the logarithmic data. This data is an average of three experimental disorder realisations.

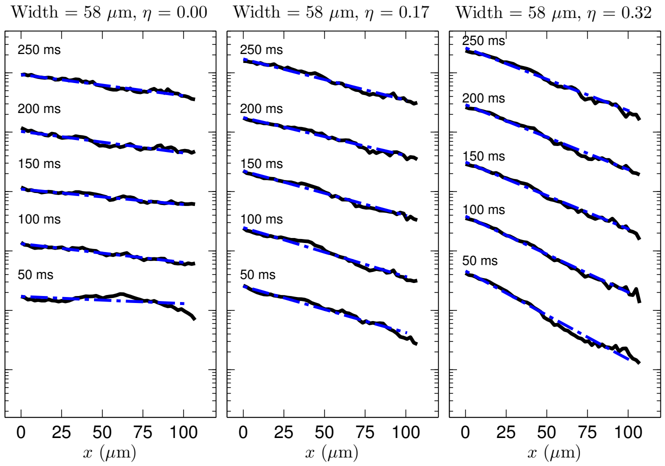

Supplementary Figure 4. 58 μm width channel density profile data. Channel profiles from 50 ms to 250 ms are plotted on a semilogarithmic scale, together with a linear fit to the logarithmic data. This data is an average of three experimental disorder realisations.

V. MOMENTUM DISTRIBUTIONS

We extend the momentum distributions plotted in Main Fig. 3 to Supplementary Fig. 9, by including the momentum distributions along the x-axis within the channel, derived from numerical simulation. (The x-axis is the direction along the channel, and a positive value means that $k_{\rm x}$ is directed towards the drain). This data also gives information regarding the direction of wave propagation, and allows us to draw conclusions regarding the scattering. The data collection time of 125 ms is chosen so as to be prior to atoms from the drain reflecting and re-entering the channel.

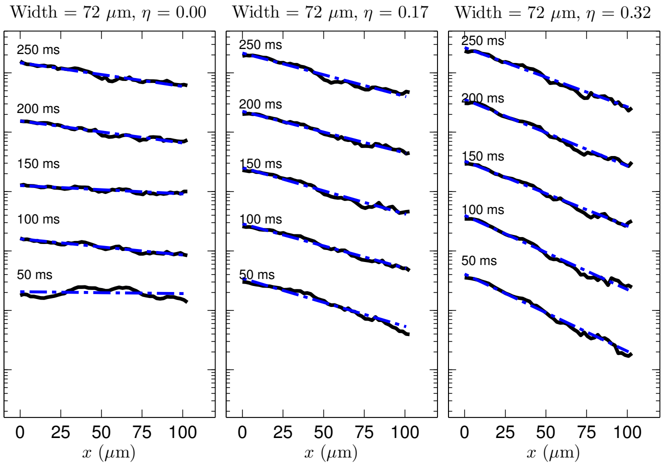

Supplementary Figure 5. 72 μm width channel density profile data. Channel profiles from 50 ms to 250 ms are plotted on a semilogarithmic scale, together with a linear fit to the logarithmic data. This data is an average of three experimental disorder realisations.

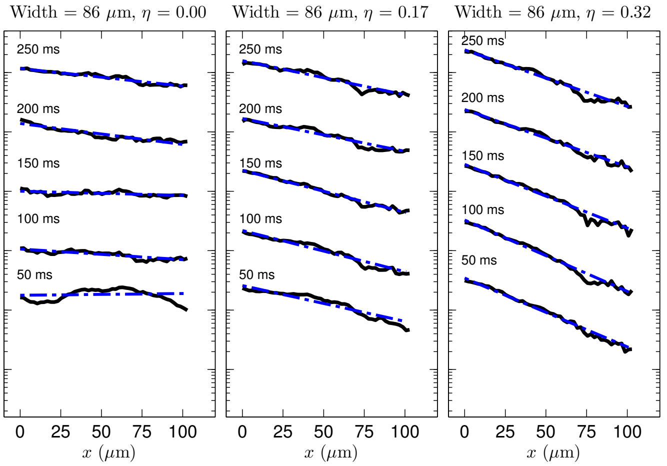

Supplementary Figure 6. 86 μm width channel density profile data. Channel profiles from 50 ms to 250 ms are plotted on a semilogarithmic scale, together with a linear fit to the logarithmic data. This data is an average of three experimental disorder realisations.

In the case of zero disorder, only atoms with a positive k_x value are present within the channel, as can be readily understood from the system geometry. In the case of disorder, scattering alters the direction of k, which smooths the k_x distribution. Moreover, we observe a bimodal distribution, consisting of a narrow distribution centred at $k_x = 0$, on top of a far broader distribution. This is further evidence that the channel contains atoms with a range of energies, with atoms in the narrow low-energy distribution experiencing exponential localisation and those in the higher energy distribution subject to weak localisation.

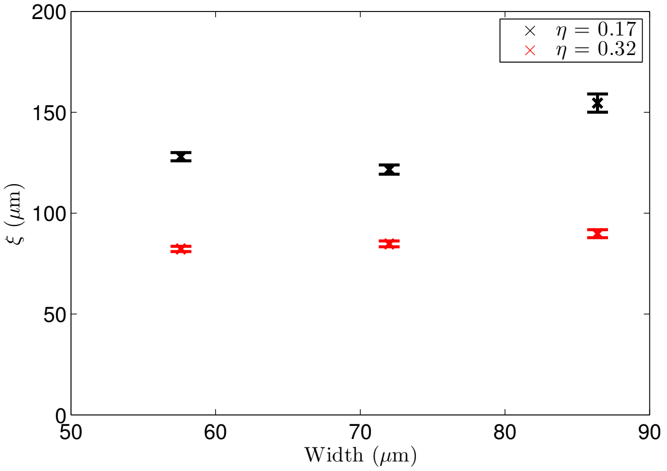

Supplementary Figure 7. Dependence of localisation length on width for a quasi-2D environment. The experimental localisation length as an average from 210-250 ms of expansion time is plotted for the width data shown in Supplementary Figs. 4-6, for $\eta = 0.17$ and $\eta = 0.32$, with $L = 108$ μm. The error bars show the standard error in the mean over the three disorder configurations.

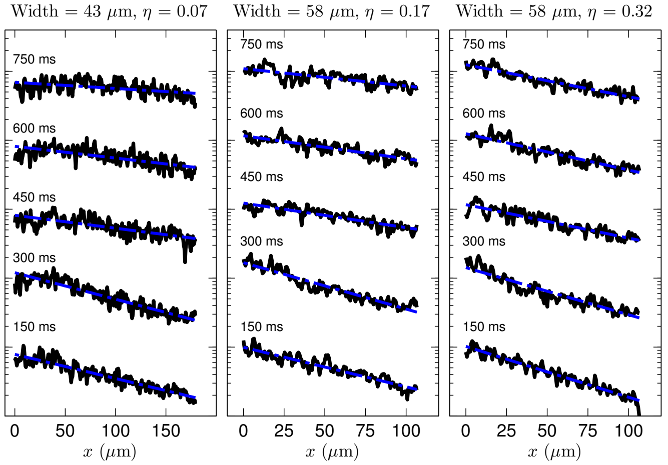

Supplementary Figure 8. Long-time simulations. Simulations for the conditions in Main Fig. 2, plotted semilogarithmically for long times. The simulations are overlaid with exponential fits to the data. Offsets by increasing factors of 10 are for clarity.

VI. POTENTIAL SLICES

Supplementary Figure 10 shows single slices of the potential applied to the spatial light modulator, as used in the experiment for three fill-factors. Each point scatterer occupies 2 pixels along the x-direction. Equivalent potentials are used for the numerical simulation. On average, a single slice will yield a fraction η of pixels within the channel of greyscale 255.

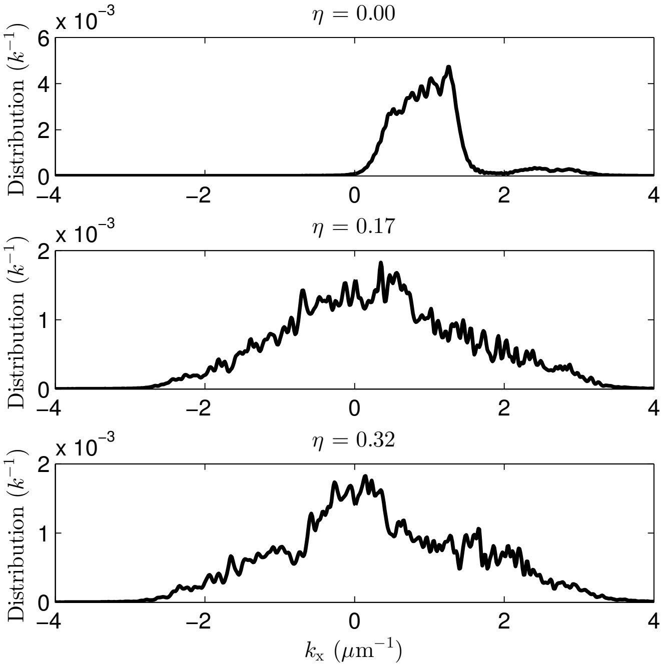

Supplementary Figure 9. Channel momentum distributions. The momentum distributions along x within the channel are obtained after 125 ms of expansion for three different fill-factors, with (r, L, w)=(43,108,58) μm, via numerical simulation.

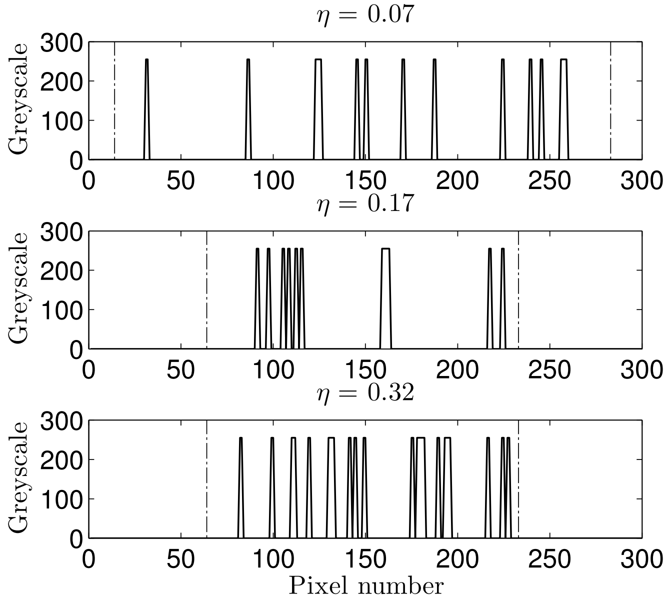

Supplementary Figure 10. Slices of dumbbell potential. Single slices of the potentials plotted in Main Fig. 2(a)-(c) are shown here, along the x-direction. Dash-dot lines indicate the mouth of the channel.

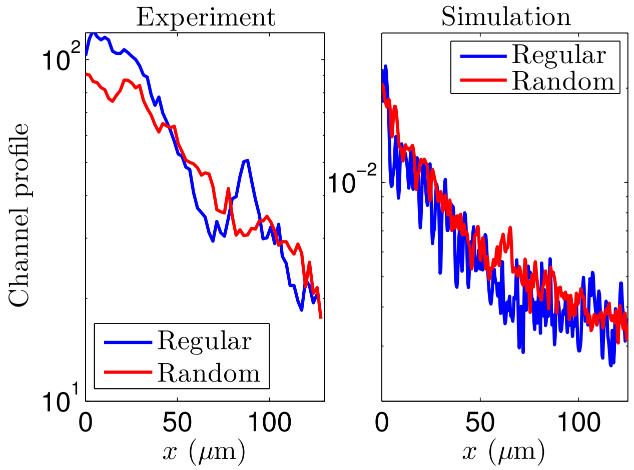

Supplementary Figure 11. Long-time density profile in regular and random lattices. (a) Experimental data. The density at fill-factor $\eta = 0.13$, $(r, L, w) = (43, 162, 43)$ μm is shown for scatterers arranged randomly, compared with scatterers arranged in a square regular lattice. (b) Numerical simulation, with equivalent parameters for (a).

VII. RESERVOIR CAPACITANCE

The channel resistance is found from the initial flow of atoms into the drain reservoir, according to

$$\frac{d\Delta N}{dt} = -\frac{\Delta N}{RC}$$

with the reservoir capacitance given by [3]

$$C = \frac{3\left(\frac{1}{2}N\right)^{1/3}}{4\alpha},$$

the constant α is

$$\alpha = \left[\frac{g\left(\frac{1}{2}m\omega_z^2\right)^{1/2}}{\frac{4}{3}\pi r^2}\right]^{2/3},$$

and $g = 4\pi\hbar^2 a_s/m$ is the 3D nonlinearity, r is the reservoir radius, m is the mass of an atom, N is the number of atoms, and ω_z is the vertical trapping frequency. In our system with $r = 43\,\mu$m, $\hbar C = 19\,$s.

VIII. COMPARISON BETWEEN ORDERED AND DISORDERED SCATTERERS

For comparison, we use the tuneability of the spatial light modulator to compare a disordered system with an ordered system, in a regime of weak localisation. In Supplementary Fig. 11(a), we compare the experimental density profiles obtained when the same number of point scatterers ($\eta = 0.13$) are arranged randomly, compared to in a regular ordered square lattice. We observe stronger linearity in the semilogarithmic plot for the disordered case. The R^2 value of the semilogarithmic profile in the regular channel is 0.90, compared with 0.95 of the disordered channel. We also point out the curvature observed in the first 20 μm in the case of regular scatterers. The increased exponential character of the

density profile in the disordered lattice is also found in the simulation (Supplementary Fig. 11(b)), and the simulation indicates that the resistance of the disordered lattice ($1.7 \times 10^{-3}h$) is 20% larger than the regular lattice ($1.4 \times 10^{-3}h$). We note the high-frequency spatial oscillation present in the simulated regular-lattice density profile, which we do not observe in the experiment due to the imaging resolution. A full study of the comparison between regularly arranged and random scatterers is currently in preparation.

IX. NUMERICAL SIMULATION

We provide the values of physical parameters used in the simulation in Supplementary Table I, and the computational parameters in Supplementary Table II. We solve the Gross-Pitaevskii equation

$$i\hbar \frac{\partial \psi(\mathbf{r},t)}{\partial t} = \left[-\frac{\hbar^2}{2m}\nabla^2_{2\mathrm{D}} + V_{\mathrm{trap}}(\mathbf{r},t) + V_{\mathrm{int}}(\mathbf{r},t)\right]\psi(\mathbf{r},t)$$

with initial condition $\psi(\mathbf{r},t) = \psi_0(\mathbf{r})$. Here $V_{\mathrm{int}}(\mathbf{r})$ denotes the interaction potential

$$V_{\mathrm{int}}(\mathbf{r}) = gN\left|\psi(\mathbf{r})\right|^2 = \frac{2\sqrt{2\pi}\,\hbar^2 a_{\mathrm{s}}}{m a_{\mathrm{z}}} N\left|\psi(\mathbf{r})\right|^2,$$

with a_{s} being the s-wave scattering length of the ^{87}Rb atoms, a_{z} is the oscillator length of the harmonic oscillator corresponding to ω_{z}. The trapping potential, $V_{\mathrm{trap}}(\mathbf{r})$, includes the strong dumbbell-shaped well of average depth V_{db}, the artificial gravitational potential and a weak harmonic potential with minimum at the centre of the dumbbell. We also note here that the presence of the linear tilt causing the artificial gravitational potential, and of the weak harmonic trap in the numerical simulation are included in the calculations for consistency with the experimental setup. The interference fringes due to distortion of the 1064 nm beam wavefront within the 2D trap are not modelled.

The initial wavefunction is the ground-state wavefunction of N interacting particles in a three-dimensional harmonic potential with angular frequencies ω_0. This ground-state wavefunction, $\psi_0(\mathbf{r})$, is determined by the standard imaginary-time propagation method [4, 5], and is shifted to the source reservoir. In most of the simulation its centre is located at the opening of the channel. However, we note that the exact position does not strongly influence the transport properties, and only moderately affect the timing. Using the adaptive, fourth-order Runge-Kutta-Fehlberg method[6] we propagate this initial wavefunction (reduced to a 2D wavefunction) in real time over a grid of size $n_{\mathrm{x}} \times n_{\mathrm{y}}$ representing the rectangular area $L_{\mathrm{x}} \times L_{\mathrm{y}}$ in real space. Here we mention that unlike the popular fourth order Runge–Kutta method (RK4), the Runge–Kutta–Fehlberg method (RKF45) is an adaptive method, i.e., it chooses the best step-size to meet a predefined error threshold. Therefore the time-step varies during in our simulations to meet the error threshold of 7×10^{-12} in L_2-norm of the wavefunction. The dumbbell –for any channel length and circular reservoir radius– is positioned at the centre of this grid symmetrically, i.e, the centre of the channel is at the centre of the numerical grid.

Supplementary Table I. Physical parameters in the numerical simulation. The various physical quantities relevant to the experiment which are used for the numerical simulation are tabulated here, together with their symbols as used in this manuscript.

Name	Symbol	Value
particle number	N	16000
^{87}Rb mass	m	$87 \times$ amu
Trap frequency (x)	ω_{x}	$2\pi \times 1$ rad/s
Trap frequency (y)	ω_{y}	$2\pi \times 1$ rad/s
Trap frequency (z)	ω_{z}	$2\pi \times 800$ rad/s
angular frequency of initial trap	ω_0	$2\pi \times 25$ rad/s
s-wave scattering length	a_{s}	$107a_0$
nonlinearity length	λ	$2\sqrt{2\pi}\,Na_{\mathrm{s}}$
angle of effective gravity	θ	$0.0002°$
potential depth	V_{db}	22 nK
scatterer strength	V_{sc}	5 nK

X. EFFECT OF INTERACTIONS

In Supplementary Fig. 12 we conduct numerical simulations for a range of interaction strengths, to determine the effect of interactions on Anderson localisation. The simulations are performed by allowing the atoms to expand from the condensate with scattering length a_{Rb}. Once the atoms have filled the first reservoir, they have acquired their initial k-vector distribution, and at this point in the simulation the interaction strength is abruptly changed to a multiple of a_{Rb}.

We observe no significant difference in the localisation lengths obtained with scattering lengths 0 and a_{Rb},

Supplementary Table II. Computational parameters in the numerical simulation. The various physical quantities relevant to the experiment which are used for the numerical simulation are tabulated here, together with their symbols as used in this manuscript.

Name	Symbol	Value
Spatial extension of the numerical grid in the x direction	L_{x}	500 μm
Spatial extension of the numerical grid in the y direction	L_{y}	225 μm
Number of grid points in the x direction	n_{x}	1536
Number of grid points in the y direction	n_{y}	768

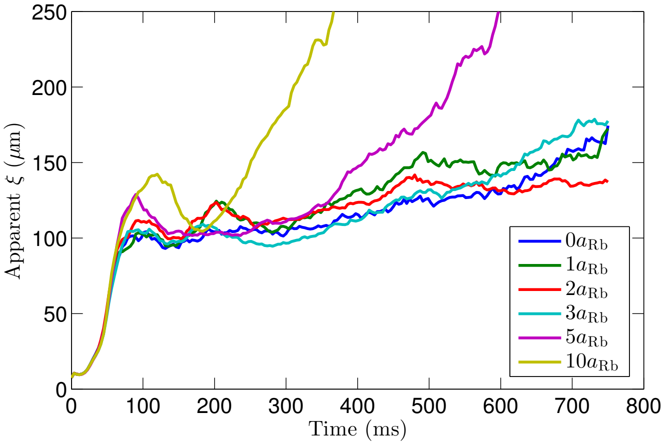

Supplementary Figure 12. Effect of interaction strength on localisation length. Apparent localisation lengths as a function of time are plotted for a range of interaction strengths, with scattering lengths ranging from 0.0–10 $a_{\rm Rb}$, as determined by numerical simulation. For this data, $(r, L, w) = (43, 108, 58)$ μm and $\eta = 0.32$.

indicating that we may view the system as near-non-interacting. This is due to the very low atomic density, averaging approximately 1 atom / μm^2.

We observe that for $a \leq 4a_{\rm Rb}$, the measured apparent localisation length tends to a steady state in time. For $a > 6a_{\rm Rb}$, we observe that the channel density profile does not tend to a steady localised state, and instead exhibits a characteristic $\xi \sim \sqrt{t}$, indicative of diffusive expansion. These results indicate that the experiment is conducted in a weakly-interacting regime in which interactions do not significantly affect Anderson localisation.

We note that the slow apparent increase in localisation length for $t > 400$ ms is due in large part to atoms which re-enter the channel from the drain reservoir, and it does not imply a weakening of Anderson localisation.

XI. RELATIONSHIP BETWEEN $\ell_{\rm s}$ AND $\ell_{\rm tr}$

The elastic mean free-path between scattering events is approximately given by the mean spacing between scatterers $\ell_{\rm s} = \sigma/\sqrt{\eta}$, while the transport mean free path, $\ell_{\rm tr}$, also known as the Boltzmann mean free path, is the distance over which the memory of the initial direction is lost. It is found [7] that $\ell_{\rm tr} = \Lambda(k\sigma)\ell_{\rm s}$, with a proportionality constant Λ dependent on relative size of a scatterer σ and the de Broglie wavelength $\lambda = 2\pi/k$. Based on the data in Main Fig. 3(e), $k\sigma \approx 1$ for the peak value of k within the channel. We emphasise that there is a distribution of atomic energies in our sample and that Λ depends strongly on $k\sigma$.

First let us determine a few characteristic quantities derived from classical or semi-classical approximations. One length scale is provided by the average momentum of atoms within the channel, as found from Main Fig. 3(e):

$$\lambda_{\rm dB} = \frac{2\pi}{k} \approx 3.9\,\mu\text{m}. \tag{1}$$

Within the channel, the de Broglie wavelength is significantly larger than the scatterer size $\sigma \approx 1.44\,\mu$m. The physical system also possesses other characteristic length scales: the length of the disordered channel $L_0 \approx 36 - 180\,\mu$m, the channel width $w \approx 14 - 87\,\mu$m, and the mean minimal distance between scatterers $\ell_{\rm s} = \sigma/\sqrt{\eta} \approx 2.5 - 3.4\,\mu$m for the corresponding fill-factors $\eta = 0.32$ and 0.17, respectively. In general, therefore, their relationship in the sequence of our experimental runs is $\sigma \lesssim \ell_{\rm s} < \lambda_{dB} \lesssim \ell_{\rm tr} < w < \xi \lesssim L_0$.

As for length scales, there are also some characteristic energy scales of this system which are given below as temperatures. Furthermore for all length scales we may associate an energy scale as well via energy $\propto \hbar^2/m(\text{length})^2$. We estimated the condensate temperature to be $T_{\rm BEC} \approx 5\,$nK, and the BEC is released in a dumbbell-shaped potential with depth of $T_{\rm pot} \approx 22\,$nK. The random scatterers have a height of $T_{\rm sc} \approx T_{\rm pot} \approx 5\,$nK. Out of these energy scales we note here the highest which corresponds to σ, the shortest length scale: $E_\sigma = \hbar^2/m\sigma^2 \approx 2.7\,$nK.

The scattering process has a decisive parameter, $k\sigma$, i.e., the relative size of the matter-wave compared to a single scatterer. Using the approximation, $k \approx k_{\rm dB}$, one obtains $k_{\rm dB}\sigma \approx 2.2$. Such value of $k\sigma$ suggests a non-isotropic scattering process even though we are still in the weak scattering regime since the atoms kinetic energies are higher than $E_{\rm sc} = \eta E_\sigma$ for all η values.

In order to establish a relationship between $\ell_{\rm tr}$ and $\ell_{\rm s}$ we evaluate Eq. (6) in Ref. [7]

$$\frac{1}{\Lambda(k\sigma)} = \frac{\ell_{\rm s}}{\ell_{\rm tr}} = 1 - \frac{\int_0^{2\pi} \cos(\theta)\mathcal{P}(k\sigma,\theta)d\theta}{\int_0^{2\pi} \mathcal{P}(k\sigma,\theta)d\theta} \tag{2}$$

where $\mathcal{P}(k\sigma,\theta) = 8\mathcal{F}(k\sigma\sin(\theta/2))$ and $\mathcal{F}(x) = \left[\arccos(x) - x\sqrt{1-x^2}\right]\Theta(1-x)$, while Θ denotes the Heaviside distribution.

Supplementary Figure 13 shows the ratio of the transport mean free path $\ell_{\rm tr}$ to the scattering mean free path $\ell_{\rm s}$, as a function of the wavenumber $|k|$. As $k\sigma \to 0^+$ the function $\mathcal{P}$ is more or less constant 4π. In this limit one may utilise that $\mathcal{F}(x) \sim \frac{\pi}{2} - 2x + \frac{1}{3}x^3$ and determines the integrals in Supplementary Eq. (2) analytically to obtain

$$\frac{\ell_{\rm tr}}{\ell_{\rm s}} \sim 1 + \frac{8}{3\pi^2}(k\sigma) + \frac{256}{9\pi^4}(k\sigma)^2 \qquad \text{as } (k\sigma) \to 0^+.$$

In the opposite limit, $k\sigma \to \infty$, the Heaviside distribution in $\mathcal{F}$ is non-zero for $0 \leq \theta \leq 2\arcsin(1/k\sigma) \approx 2/k\sigma$ or $2\pi - 2/k\sigma \leq \theta \leq 2\pi$. Therefore the bounds of integrals in Supplementary Eq. (2) are also restricted to these two small intervals. However, the few leading terms in the Taylor expansion of $\mathcal{F}$ are not sufficient to determine the asymptotic behaviour of $\Lambda(k\sigma)$, but one needs to retain

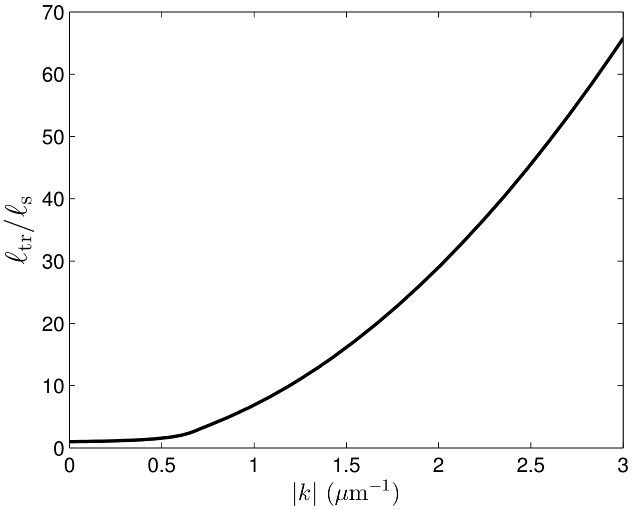

Supplementary Figure 13. **Λ as a function of wavenumber.** The ratio of the transport mean free path $\ell_{\rm tr}$ to the scattering mean free path $\ell_{\rm s}$ is plotted as a function of $|k|$, where σ is assumed to be 1.4 μm. For $|k| = 1.0\ \mu\text{m}^{-1}$, $\ell_{\rm tr} \approx 7\ell_{\rm s}$.

all terms in the expansion

$$\mathcal{F}(x) = \frac{\pi}{2} - 2x + \sum_{n=1}^{\infty} \frac{1}{2^{2n-1}(2n-1)(2n+1)} \binom{2n}{n} x^{2n+1}.$$

The leading term of $\Lambda(k\sigma)$ is quadratic, $c_2(k\sigma)^2$, where the explicit expression of c_2 cannot be given in finite terms. Its value is approximately

$$c_2 \cong \frac{4}{3}\,\frac{6\pi - 11}{6\pi - 16} \approx 3.673$$

At the end we may evaluate the analytic first order correction [7] to the Boltzmann diffusion coefficient for an indicative value of the fill-factor, $\eta = 0.06$,

$$\frac{\delta D}{D_{\rm B}} = \frac{2}{\pi}\,\frac{\ln(L_0/\ell_{\rm s})}{k\ell_{\rm tr}} \approx 3.6 \times 10^{-3}.$$

REFERENCES

[1] D. S. Petrov, M. Holzmann, and G. V. Shlyapnikov, "Bose-Einstein condensation in quasi-2D trapped gases," Phys. Rev. Lett. **84**, 2551–2555 (2000).

[2] Peter Krüger, Zoran Hadzibabic, and Jean Dalibard, "Critical point of an interacting two-dimensional atomic Bose gas," Phys. Rev. Lett. **99**, 040402 (2007).

[3] Aijun Li, Stephen Eckel, Benjamin Eller, Kayla E. Warren, Charles W. Clark, and Mark Edwards, "Superfluid transport dynamics in a capacitive atomtronic circuit," Phys. Rev. A **94**, 023626 (2016).

[4] Weizhu Bao and Qiang Du, "Computing the ground state solution of Bose–Einstein condensates by a normalized gradient flow," SIAM J. Sci. Comput. **25**, 1674–1697 (2004).

[5] Wilhelm Magnus, "On the exponential solution of differential equations for a linear operator," Commun. Pure Appl. Math **7**, 649–673 (1954).

[6] R.L. Burden and J.D. Faires, *Numerical Analysis* (Brooks/Cole, Cengage Learning, 2011).

[7] R. C. Kuhn, C. Miniatura, D. Delande, O. Sigwarth, and C. A. Müller, "Localization of matter waves in two-dimensional disordered optical potentials," Phys. Rev. Lett. **95**, 250403 (2005).
